# Chloride-guided bolus vs conventional fluid therapy for preoperative optimisation in infantile hypertrophic pyloric stenosis

**DOI:** 10.1308/rcsann.2025.0061

**Published:** 2026-01-20

**Authors:** MS Saleem, U Mahmood, M Rehan, CE Azmat

**Affiliations:** University of Child Health Sciences, The Children’s Hospital, Lahore, Pakistan

**Keywords:** Infantile hypertrophic pyloric stenosis, Fluid therapy, Electrolytes, Randomised controlled trial

## Abstract

**Introduction:**

Infantile hypertrophic pyloric stenosis (IHPS) often presents with significant metabolic derangement requiring preoperative fluid resuscitation. Conventional fluid therapy (CFT) is widely used, but bolus fluid therapy (BFT), guided by serum chloride levels, may allow faster correction and improved outcomes. This study compared the safety and efficiency of BFT vs CFT in infants with IHPS.

**Methods:**

A single-centre randomised controlled trial was conducted over 30 months at a tertiary paediatric surgical unit in Pakistan. Infants aged 2–12 weeks with confirmed IHPS were randomly assigned to receive either CFT or BFT. CFT involved maintenance fluids with potassium supplementation and 6-hourly monitoring. BFT comprised 20ml/kg saline boluses tailored by initial chloride and bicarbonate levels, based on the Dalton algorithm, with monitoring before and after each bolus. Primary outcomes included time to biochemical optimisation, hospital stay, and number of laboratory tests.

**Results:**

One hundred infants were enrolled (*n* = 50 per group). The BFT group achieved faster correction (7.1 ± 2.2h vs 71.5 ± 10.3h; *p* = 0.001), shorter hospital stay (118.6 ± 29.9h vs 154.5 ± 37.3h; *p* = 0.001), and fewer laboratory tests (3.2 ± 0.9 vs 4.8 ± 1.1; *p* = 0.02). No complications occurred.

**Conclusions:**

Chloride-guided BFT is a safe, efficient alternative to CFT for IHPS. It reduces time to correction, length of stay and investigation burden. Early discharge may also reduce nosocomial risk, offering particular benefit in resource-limited settings.

## Introduction

Infantile hypertrophic pyloric stenosis (IHPS) is a common condition in early infancy that frequently necessitates surgical intervention. First described by Harald Hirschsprung in 1888, IHPS was characterised by pyloric muscle hypertrophy and gastric outlet obstruction in two infants examined post mortem.^1,2^

The condition typically presents between 2 and 12 weeks of age, manifesting as projectile, non-bilious vomiting. This often leads to significant dehydration and a characteristic biochemical profile comprising hypochloraemic, hypokalaemic metabolic alkalosis.^3^

Ramstedt pyloromyotomy, introduced in 1911–1912, remains the most widely used surgical treatment for IHPS.^3,4^ However, non-operative management using atropine has also been reported, particularly in settings where surgery may not be immediately feasible or is contraindicated.^5,6^ It is now widely accepted that surgical intervention is not truly urgent, and may be safely delayed until adequate metabolic resuscitation has been achieved. Many clinicians regard base excess as the most reliable marker of preoperative metabolic correction, although serum chloride and bicarbonate remain useful adjuncts.^7^

In cases in which electrolyte disturbances are evident at diagnosis – specifically chloride levels below 100mmol/l, bicarbonate levels above 30mmol/l or potassium outside the range of 3.4–5.2mmol/l – careful intravenous fluid resuscitation is required prior to surgery. Operating is generally considered safe once chloride exceeds 100mmol/l, bicarbonate falls below 30mmol/l and base excess returns to near-normal values.^7^ Persistent metabolic derangement on presentation is associated with prolonged hospitalisation, increased fluid requirements and more frequent laboratory testing.^3^ Accordingly, standardised protocols for fluid optimisation are increasingly recognised as a means to reduce delays in surgical treatment.^8^

In the past decade, two principal strategies for preoperative resuscitation have been evaluated. Conventional fluid therapy (CFT), typically comprising 5% dextrose in 0.45% saline with potassium supplementation at 1.25–2 times maintenance, aims to achieve metabolic stabilisation within 24h.^3^ Alternatively, bolus fluid therapy (BFT) employs weight-based saline boluses guided by defined serum electrolyte thresholds. Emerging evidence suggests that BFT may result in more rapid correction of metabolic disturbances and shorter hospital stays.^9^

Given the substantial burden of IHPS in low- and middle-income countries (LMICs) – estimated at approximately 2 per 1,000 live births – the identification of an optimal, resource-efficient preoperative fluid management strategy is of considerable importance.^10^ This randomised controlled trial was designed to compare chloride-guided BFT with CFT, with the aim of evaluating efficiency and safety in the preoperative optimisation of infants with IHPS, particularly in the context of resource-constrained healthcare environments.

## Methods

A single-centre, randomised controlled trial (UTN#U1111 1232 8922) was conducted over a 30-month period in the Department of Paediatric Surgery at the University of Child Health Sciences and Children's Hospital, Lahore, Pakistan. Ethical approval was obtained from the institutional review board on 28 March 2018. Written informed consent was obtained from the parents or legal guardians of all participants prior to enrolment and randomisation.

Infants aged between 2 and 12 weeks with clinically and sonographically confirmed IHPS were eligible for inclusion. Exclusion criteria included the presence of congenital heart disease, renal failure, sepsis or any other significant comorbidities. In total, 100 infants meeting the inclusion criteria were enrolled and randomly allocated into two equal groups (*n* = 50 each) using a sealed-envelope lottery method following consent. Group A received CFT, and group B received BFT.

In the CFT group, maintenance fluids were administered as 5% dextrose in 0.45% saline at 1.25–2 times the standard maintenance rate. Potassium supplementation was added based on urinary output. Arterial blood gases and serum electrolytes were monitored every 6h until biochemical normalisation.

In the BFT group, maintenance fluids were administered at 1.5 times the standard rate in addition to 20ml/kg boluses of normal saline. The number of boluses was determined according to initial biochemical parameters: three boluses were given for chloride levels below 85mmol/l, two boluses for chloride between 85 and 97mmol/l and one bolus for chloride between 97 and 99mmol/l. In cases with normal chloride but bicarbonate >33mmol/l, two to three boluses were administered. If both chloride and bicarbonate were normal but potassium was <3.5mmol/l, a single bolus was given. Arterial blood gases and electrolyte levels were checked before and 1h after each bolus, and further boluses were administered based on follow-up results.

This chloride-based strategy was guided by the Dalton algorithm for preoperative optimisation in IHPS, which recommends estimating chloride requirements based on presenting electrolyte derangements. According to the algorithm, approximately 10mmol/kg of chloride is required to reduce plasma bicarbonate by 3mmol/l, thus allowing for tailored correction of metabolic alkalosis through calculated saline boluses.^11^ This approach was used to individualise fluid resuscitation and expedite correction while maintaining patient safety.

Sample size was determined using EpiTools, assuming a 95% confidence level, 90% statistical power and an expected difference in hospital stay based on data reported by Dalton *et al* (2.6 vs 1.9 days).^11^ A 15% attrition rate was accounted for in the calculation. Although prevalence-based sample calculations are more commonly applied in observational research, this pragmatic design ensured adequate enrolment to detect meaningful clinical effects.

### Statistical analysis

All clinical data, including fluid administration, hydration status (assessed via skin turgor, fontanelle tension, mucous membrane moisture and urine output), bolus frequency and serial laboratory parameters, were prospectively recorded by the principal investigator. Data analysis was performed using SPSS version 27. Continuous variables were expressed as mean ± sd and categorical variables as counts and percentages. As per CONSORT guidelines for randomised controlled trials, only post-intervention outcomes such as time to biochemical optimisation and hospital stay were subjected to inferential statistical testing using independent sample *t*-tests, with significance defined as *p* < 0.05. Baseline characteristics were summarised descriptively without statistical comparison. The statement regarding the absence of complications is reported in the Results section in accordance with standard reporting practices.

## Results

A total of 100 infants diagnosed with IHPS were enrolled and successfully completed the study protocol following random allocation, with 50 patients in each group. No complications were reported in either cohort.

Baseline demographic characteristics, including age, sex, weight, duration of symptoms prior to presentation, and admission biochemical parameters, were comparable between the two groups and are summarised in Table 1. In line with CONSORT guidelines for randomised trials, no statistical comparisons were applied to these baseline data.

**Table 1:** Primary postintervention outcomes

Outcome	Conventional fluid therapy (*n*=50) Mean ± sd	Bolus fluid therapy (*n* = 50) Mean ± sd	*p*-value
Time to biochemical optimisation (h)	71.5 ± 10.3	7.1 ± 2.2	**0.001**
Total hospital stay (h)	154.5±37.3	118.6±29.9	**0.001**
Laboratory tests per patient (ABG/BMP)	4.8±1.1	3.2±0.9	**0.02**

Lab-test frequency includes all arterial blood gases (ABG) and electrolyte panels obtained during preoperative optimisation. BMP = basic metabolic panel

Significant differences were observed in post-intervention outcomes following fluid resuscitation. The mean time to achieve biochemical optimisation was markedly shorter in the BFT group at 7.1 ± 2.2h, compared with 71.5 ± 10.3h in the CFT group (*p* = 0.001). Likewise, the mean total hospital stay was significantly reduced in the BFT group, at 118.6 ± 29.9h compared with 154.5 ± 37.3h in the CFT group (*p* = 0.001). The BFT group also underwent fewer laboratory investigations, averaging 3.2 ± 0.9 arterial blood gas or basic metabolic panel assessments per patient, vs 4.8 ± 1.1 in the CFT group (*p* = 0.02), as detailed in Table 1.

Further descriptive data regarding the duration of conventional therapy are shown in Table 2. Among infants receiving CFT, 8% completed fluid resuscitation within 48h, whereas the remaining 92% required up to 72h. The number of saline boluses administered in the BFT group is presented in Table 3. Of these infants, 18% received one bolus, 44% received two boluses, 26% received three boluses and 12% required four boluses. All bolus administration followed strict adherence to the predefined algorithm based on serum chloride and metabolic parameters.

**Table 2:** Timing of conventional fluid therapy (CFT) completion

Timing of CFT completion	Number (%)
Within 48 h	4 (8.0)
Within 72 h	46 (92.0)

**Table 3:** Number of boluses administered in bolus fluid therapy

Number of boluses administered	Number (%)
1	9 (18.0)
2	22 (44.0)
3	13 (26.0)
4	6 (12.0)

Although a greater number of boluses were often required in infants presenting later, this reflects the greater biochemical derangement at admission and does not indicate deviation from the randomisation protocol. Overall, these results indicate that BFT led to significantly faster biochemical correction, shorter hospitalisation and reduced laboratory monitoring, without any adverse effects.

## Discussion

This randomised controlled trial demonstrates that chloride-guided BFT significantly reduces the time to biochemical optimisation and total hospital stay compared with CFT in infants with IHPS. The findings suggest that BFT, when guided by initial electrolyte status and implemented within a structured algorithm, can be an effective and efficient preoperative resuscitation strategy in IHPS.

Although CFT has long been considered the default fluid management strategy, its limitations are evident – particularly the prolonged time to achieve metabolic correction and readiness for surgery.^3,4,7^ Our study supports emerging evidence that protocolised BFT expedites biochemical recovery without adverse effects, thereby reducing the need for prolonged hospitalisation and excessive laboratory monitoring. These outcomes are aligned with those from prior studies that implemented structured care pathways or standardised fluid protocols and reported similar benefits, including shorter lengths of stay and improved parental satisfaction.^12–14^

Chloride remains a particularly sensitive and practical biomarker in guiding fluid resuscitation in IHPS, as also highlighted in previous research.^15,16^ Our algorithm adjusted bolus volume and frequency based on the degree of hypochloraemia and metabolic alkalosis. This approach is supported by earlier work indicating that approximately 10mmol/kg of chloride is needed to reduce plasma bicarbonate by 3mmol/l.^15^ The correlation between initial chloride levels and the required volume and timing of resuscitation highlights the clinical utility of a chloride-based algorithm.

Importantly, the streamlined BFT approach also translated into fewer blood draws per patient, reinforcing its role in reducing procedural burden – a factor often overlooked in neonatal care but essential for enhancing parental satisfaction and minimising iatrogenic complications.^13,14^ These results are clinically meaningful, particularly in low-resource settings, although the benefits may be generalisable across healthcare systems.

### Study limitations

Despite these strengths, our study has limitations. It was conducted at a single tertiary-care centre in a LMIC, which may affect external validity. However, the relatively large sample size and adherence to a uniform protocol strengthen internal validity. Moreover, our findings resonate with earlier reports from high-income settings, suggesting the broader applicability of this approach.^13,14,17^

The trial also addresses an important gap: the lack of standardised, evidence-based preoperative resuscitation protocols in IHPS. Although atropine-based non-operative treatment remains an option in selected settings, surgical management following adequate fluid correction continues to be the most widely accepted practice.^3–6^ Our results support the adoption of structured, chloride-guided BFT protocols to improve preoperative readiness and reduce the burden of care.

Early discharge following metabolic optimisation not only shortens total hospital stay, but also reduces the infant's exposure to nosocomial pathogens. In resource-constrained settings where infection control infrastructure may be limited, this aspect of care is particularly relevant. Our findings, therefore, support BFT not only as a means of clinical efficiency, but also as a strategy to minimise iatrogenic risks associated with prolonged inpatient stay.

## Conclusions

This randomised controlled trial establishes that chloride-guided BFT offers a safe and superior approach to preoperative optimisation in infants with hypertrophic pyloric stenosis. By significantly reducing the time to correction, hospital stay and investigative burden, this strategy presents a clinically effective alternative to conventional protocols. Its structured, electrolyte-based design lends itself well to both high- and low-resource settings, supporting its integration into standard paediatric surgical practice.
